# High-quality Janus MoSSe and WSSe monolayers enabled by hBN-supported chalcogen substitution

**DOI:** 10.1080/14686996.2026.2713989

**Published:** 2026-08-07

**Authors:** Tomoya Ogawa, Wenjin Zhang, Takahiko Endo, Yuta Sawai, Dingkun Bi, Tianyishan Sun, Hiroto Ogura, Kenji Watanabe, Takashi Taniguchi, Toshiaki Kato, Yasumitsu Miyata

**Affiliations:** a Department of Physics, Tokyo Metropolitan University, Hachioji, Japan; b Research Center for Materials Nanoarchitectonics (MANA), National Institute for Materials Science (NIMS), Tsukuba, Japan; c Advanced Institute for Materials Research (AIMR), Tohoku University, Sendai, Japan; d Graduate School of Engineering, Tohoku University, Sendai, Japan; e Research Center for Electronic and Optical Materials, NIMS, Tsukuba, Japan

**Keywords:** 2D materials, transition metal dichalcogenides, Janus, MoSSe, WSSe, chemical vapor deposition, optical properties

## Abstract

Janus transition metal dichalcogenide (TMD) monolayers offer distinctive physical properties and device applications because their broken out-of-plane mirror symmetry induces an intrinsic out-of-plane dipole. High crystal quality is essential for accessing their intrinsic excitonic physics, yet improving Janus TMD quality remains challenging due to structural degradation and strain introduced during chalcogen substitution. To address this challenge, we develop a hexagonal boron nitride (hBN)-supported chalcogen substitution process that yields crack-free Janus TMD monolayers with improved optical uniformity. MoSe_2_ and WSe_2_ monolayers are first grown on hBN substrates and then converted into Janus MoSSe and WSSe monolayers via room-temperature H_2_ plasma treatment, respectively. Compared with conventional SiO_2_-supported samples, the hBN-supported process yields Janus monolayers with reduced inhomogeneous lattice strain, owing to negligible in-plane interactions with the substrate. The hBN-supported samples exhibit narrow photoluminescence (PL) linewidths of 40–50 meV at room temperature, which further decrease to ~9 meV at 5 K. This linewidth reduction enables quantitative analysis of the trion binding energy and exciton–phonon interaction and provides a practical route to accessing intrinsic properties of Janus TMDs.

## IMPACT STATEMENT

Janus two-dimensional semiconductors have different atoms on their top and bottom surfaces, giving them unique electrical and optical properties. However, making high-quality Janus layers is difficult because the atomic replacement process can damage the material and introduce strain. This work shows that using atomically flat hBN as a support suppresses cracking and disorder during Janus conversion, producing more uniform Janus MoSSe and WSSe monolayers. This approach will help reveal their intrinsic light-emission properties and support future development of Janus-based nanoscale devices.

## GRAPHICAL ABSTRACT

**Figure:**
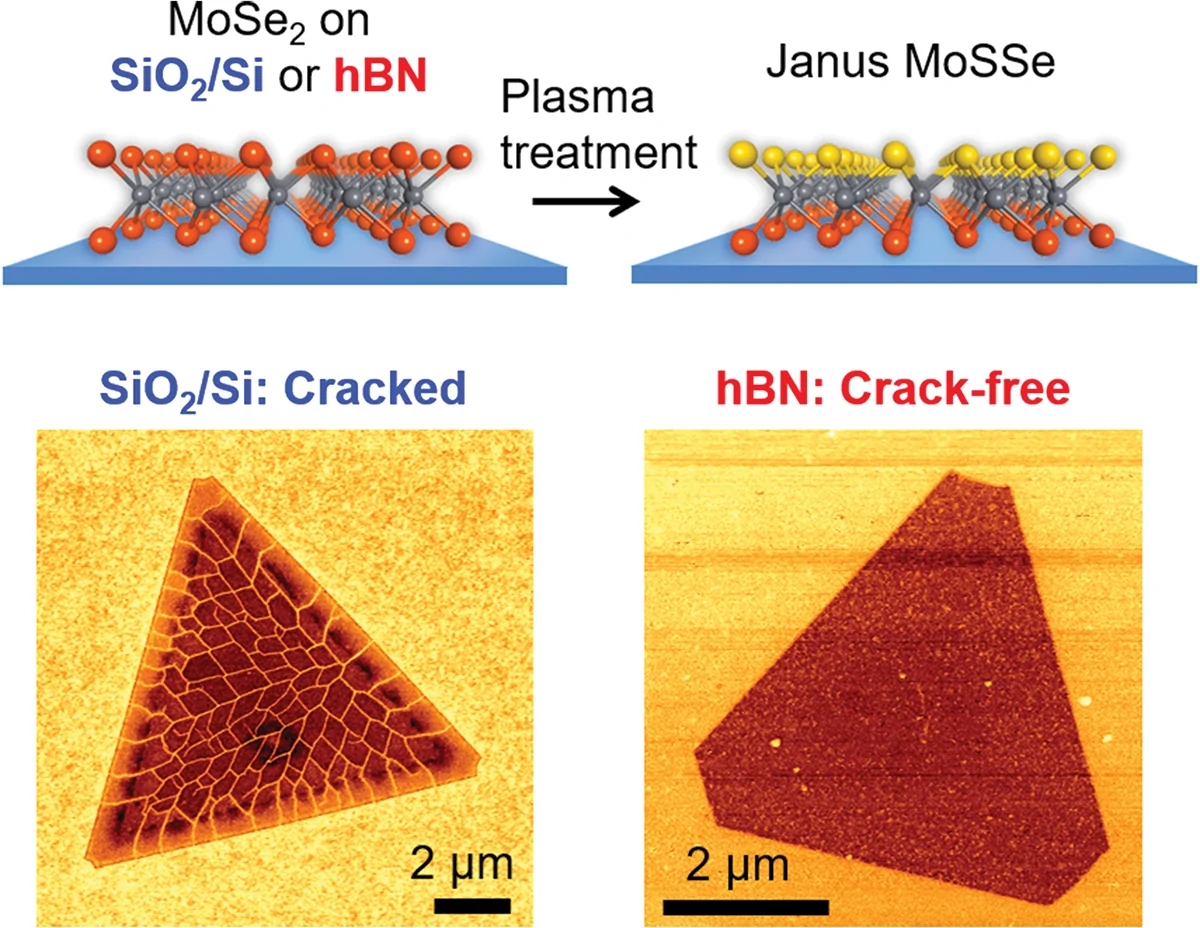

## 1. Introduction

Transition metal dichalcogenides (TMDs) have attracted considerable attention because of their distinctive physical properties and potential device applications. Monolayer TMDs consist of a transition metal layer sandwiched between two chalcogen layers and are commonly denoted as MX_2_ (*M* = Mo, W; X = S, Se, Te). Due to their crystal structure containing heavy atoms and broken inversion symmetry, TMD monolayers exhibit spin-valley coupled band structures and strong light-matter interactions [1]. To further expand their functionalities, systems with reduced crystal symmetry have attracted increasing interest. In this context, Janus TMD monolayers, where the top and bottom chalcogen species differ, have emerged as a prominent platform. In particular, their out-of-plane asymmetry produces an intrinsic dipole (built-in electric field) [2], enabling a range of phenomena including long-lived charge-transfer excitons [3–5], the Rashba effect [2,6,7], piezoelectricity [8], enhanced catalytic activity [9], and dipole-assisted reduction of contact resistance [10].

Since the first experimental report of Janus TMDs in 2017, fabrication methods have been continuously refined. Janus TMDs were initially synthesized by two different methods [11,12]: (i) H_2_ plasma treatment followed by thermal selenization of the top surface of monolayer MoS_2_ [11], and (ii) thermal sulfurization of the top surface of monolayer MoSe_2_ [12]. More recently, room-temperature plasma-based conversion has emerged as a promising alternative, offering improved material quality [13–16]. These advances further expand the scope of Janus TMDs, enabling the exploration of their physical properties [4,17–23] and the realization of new nanostructures including moiré superlattices [24] and nanoscrolls [25–28]. Nevertheless, Janus TMDs converted from CVD-grown TMDs on SiO_2_/Si substrates still typically exhibit lower crystallinity than those obtained from exfoliated crystals [15]. This discrepancy is often attributed to the higher defect densities and/or residual lattice strain in CVD-grown monolayers on SiO_2_/Si. However, the key factors governing crystallinity preservation during chalcogen substitution remain poorly understood.

In general, lattice strain is readily introduced in atomically thin TMDs, strongly influencing their stability and chemical reactivity. In CVD-grown TMD monolayers on SiO_2_, non-uniform strain can develop during cooldown because of the mismatch in thermal expansion coefficients between the substrate and the TMD [29–32]. During chalcogen substitution, the lattice constant changes by ~2% due to the different ionic radii of the chalcogen atoms [24]. When the TMD-substrate interaction is strong, the chalcogen substitution can generate additional strains and even induce cracking. Conversely, TMDs grown on van der Waals substrates such as hBN and graphite typically exhibit minimal residual strain, because atomically flat surfaces and weak interfacial pinning facilitate strain relaxation [30]. Consistently, TMD monolayers grown on hBN often show very narrow photoluminescence (PL) linewidths. These considerations suggest that hBN is an ideal platform for fabricating high-quality Janus TMD monolayers via chalcogen substitution.

In this study, we report the preparation of high-quality Janus MoSSe and WSSe monolayers by room-temperature H_2_ plasma treatment of CVD-grown MoSe_2_ and WSe_2_ monolayers on hBN. The crystallinity and uniformity of the resulting Janus monolayers were evaluated by PL and Raman spectroscopy, together with Kelvin probe force microscopy (KPFM). KPFM revealed crack-free Janus monolayers on hBN, in stark contrast to the extensive cracking observed for samples converted on SiO_2_/Si. PL and Raman measurements further confirmed uniform conversion of entire MoSe_2_ and WSe_2_ grains into the Janus structure, accompanied by suppressed non-uniform strain across the grains. Finally, we investigated the excitonic properties of the Janus monolayers using temperature-dependent PL spectroscopy.

## 2. Experimental method

### 2.1. CVD growth of monolayer TMDs

**Figure 1.**
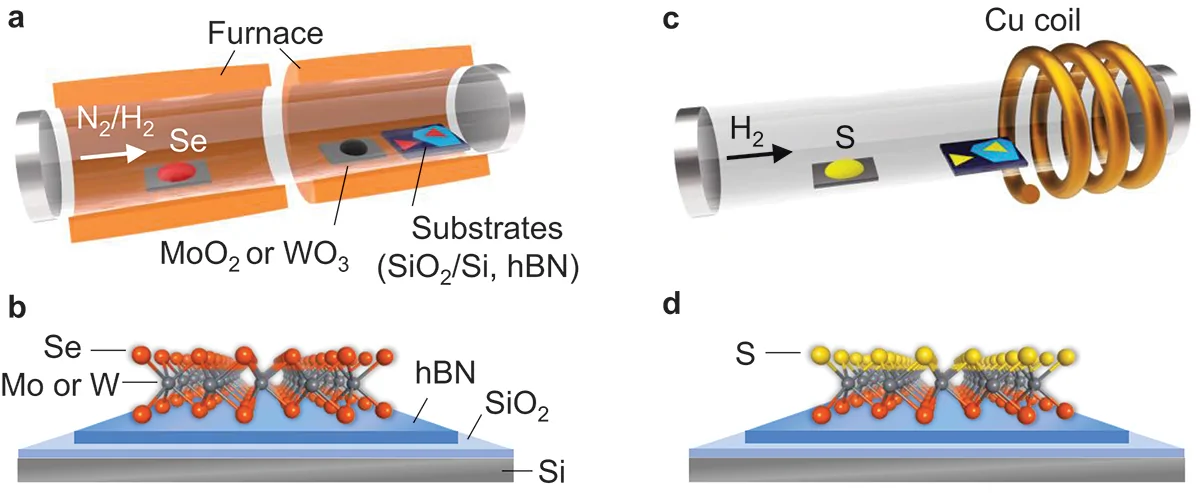
Preparation of TMD and Janus TMD monolayers. Schematics of (a) the CVD setup, (b) a MoSe_2_ (or WSe_2_) monolayer grown on an hBN flake on an SiO_2_/Si substrate, (c) the H_2_ plasma treatment system, and (d) a Janus MoSSe (or WSSe) monolayer obtained after H_2_ plasma treatment.

Monolayer MoSe_2_ and WSe_2_ were grown by chemical vapor deposition (CVD) on SiO_2_/Si substrates (285 nm of SiO_2_) and on hBN substrates (Figure 1(a,b)), as reported previously [33]. hBN flakes were mechanically exfoliated from bulk crystals [34] and transferred onto SiO_2_/Si substrates. For MoSe_2_, MoO_2_ powder (80–120 mg) and Se beads (2–4 g) were loaded in a quartz tube together with the growth substrate. The tube was purged and then maintained at atmospheric pressure under a mixed flow of N_2_ (200–250 sccm) and H_2_ (1 sccm). The MoO_2_ and Se were heated to 870–880°C and 420°C, respectively, using the downstream and upstream electric furnaces. After reaching the target temperature, the system was held for 2 min and then cooled to room temperature using an electric fan. For WSe_2_ growth, WO_3_ powder (300 mg) and Se beads (3–4 g) were used as precursors. A diluted H_2_/N_2_ (H_2_ = 0.7%) gas was flowed at a total rate of 450 sccm. The furnace temperatures were set to 950°C for WO_3_ and 380°C for Se.

#### 2.1.1. H_2_ plasma-assisted surface atomic substitution

Janus monolayers on SiO_2_/Si and hBN substrates were prepared by plasma-assisted surface chalcogen substitution starting from monolayer TMDs (Figure 1(c,d)) [13,15,24,26,27]. CVD-grown TMD samples (MoSe_2_ and WSe_2_) were placed in a quartz tube (inner diameter, 20 mm; length, 600 mm) at *z* = 300–350 mm, where *z* = 0 mm denotes the upstream end of the quartz tube. The tube was evacuated to a few Pa, and then supplied with 99.99% H_2_ at ~20 sccm, while the tube pressure was maintained at ~38 Pa. Sulfur powder was placed upstream at *z* = 200–250 mm. A copper coil (diameter, 50 mm) was wrapped around the downstream region (z = 400–500 mm) as an antenna for inductively coupled plasma generation. The radio-frequency power applied to the copper coil was set to 15–30 W, and the reaction time was 30–60 min. All treatments were carried out at room temperature.

#### 2.1.2. Transfer process

For the hBN encapsulation, we used a polymer-assisted dry-transfer method reported previously [35]. A transfer stamp was prepared by drop-casting an acrylic resin solution (Elvacite 2552C in anisole) onto a glass slide and baking it on a hot plate at 185°C for 20 min. The glass slide (stamp) and an exfoliated hBN flake on an SiO_2_/Si substrate were mounted on a transfer setup consisting of xyz stages, a hot plate, and an optical microscope. To pick up the hBN, the stamp was brought into contact with the hBN flake, heated to 160°C to promote adhesion, and then cooled to 50°C using an electric fan. The hBN flake was picked up by retracting the glass slide. Subsequently, the Janus TMD/hBN sample was picked up with the top hBN using the same procedure. The stamp carrying the stack was then brought into contact with a fresh SiO_2_/Si substrate and heated to 185°C to release the stack by dissolving the acrylic resin. The remaining polymer was removed by rinsing with chloroform, yielding hBN-encapsulated Janus TMD.

#### 2.1.3. Characterizations

Local surface-potential maps were acquired by Kelvin probe force microscopy (KPFM) in noncontact mode using an atomic force microscope (NX10, Park Systems, Republic of Korea). Room temperature PL and Raman measurements were carried out using a Renishaw inVia confocal system (Renishaw plc, UK) equipped with a 532 nm excitation laser. Cryogenic PL spectra were obtained using a home built optical setup with a 532 nm excitation laser and a a cryogen-free cryostat (Cryo Core, Montana Instruments, USA). The excitation laser was focused using a 100x objective lens, and the emitted signal was collected by the same objective and recorded with a spectrometer equipped with an EMCCD detector.

## 3. Results and discussion

**Figure 2.**
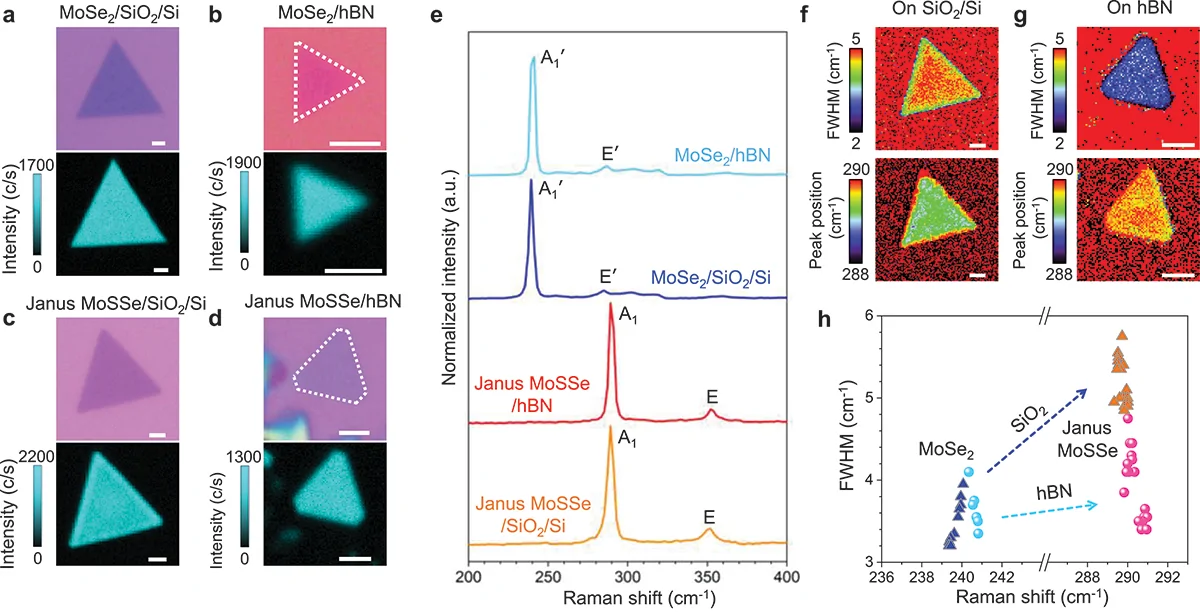
Raman characterization of MoSe_2_ and Janus MoSSe monolayers. (a-d) Optical micrographs and corresponding Raman intensity maps of (a,b) the A_1_’ mode of MoSe_2_ at 240 cm^−1^ and (c,d) the A_1_ mode of Janus MoSSe at 290 cm^−1^ on SiO_2_/Si and hBN. (e) Representative Raman spectra of MoSe_2_ and Janus MoSSe monolayers on SiO_2_/Si and hBN. (f,g) Spatial maps of the FWHM and peak position of the A_1_ mode for Janus MoSSe on (f) SiO_2_/Si and (g) hBN. (h) Distributions of the FWHM and peak positions of the A_1_’ mode of MoSe_2_ and the A_1_ mode of Janus MoSSe on SiO_2_/Si and hBN. All scale bars are 2 µm.

The formation of Janus MoSSe and WSSe monolayers was initially identified by Raman spectroscopy. We note that Raman spectra provide a rapid check of one-sided chalcogen substitution through Raman signatures distinct from those of random MoS_2(1-*x*)_Se_2*x*_ and WS_2(1-*x*)_Se_2*x*_ alloys [11,12,23,36]. In addition, one-sided chalcogen substitution has been directly observed by scanning transmission electron microscopy in previous studies [11,14,26,37], including our own work. The monolayer nature was also confirmed by strong PL peaks as shown later. Figure 2(a–d) shows optical micrographs and Raman maps of MoSe_2_ and Janus MoSSe monolayers prepared on SiO_2_/Si and hBN. As shown in the Raman spectra (Figure 2(e)), MoSe_2_ exhibits an A_1_’ mode at ~240 cm^−1^ and an E’ mode at ~286 cm^−1^, while Janus MoSSe shows the characteristic A_1_ mode at ~290 cm^−1^ and an E mode at ~352 cm^−1^ [36]. The Raman intensity maps confirm uniform conversion of the entire MoSe_2_ grains into Janus MoSSe monolayer (Figure 2(a–d)).

To assess the crystallinity and uniformity, we analyzed the peak frequencies and full width at half-maximum (FWHM) of the A_1_’/A_1_ modes (Figure 2(f,g)). Janus MoSSe on hBN shows a narrower FWHM over the entire grain than that on SiO_2_/Si, and its A_1_-mode peak is slightly blueshifted. Figure 2(h) summarizes the distributions of the A_1_’/A_1_-mode FWHM and peak frequency obtained from multiple MoSe_2_ and Janus MoSSe monolayers on each substrate. Overall, Janus MoSSe on hBN shows a pronounced linewidth reduction, together with a slight increase in the A_1_-mode peak frequency. Notably, the minimum FWHM for Janus MoSSe on hBN reaches 3.4 cm^−1^, which is smaller than that for Janus MoSSe obtained from mechanically exfoliated MoSe_2_ (~3.8 cm^−1^) [15]. Similarly, WSe_2_ monolayers are successfully converted into Janus WSSe, and the resulting WSSe monolayers on hBN show a reduced linewidth down to 4 cm^−1^ with a narrower distribution of FWHM values (Figure S1, Supplementary Information).

The observed linewidth narrowing for Janus MoSSe and WSSe can be attributed to substrate-dependent strain relaxation. CVD-grown TMDs on SiO_2_/Si often experience tensile strain owing to the mismatch in thermal expansion coefficients and strong interfacial interactions [29–32]. Such tensile strain softens the Raman mode, leading to a redshift of the peak frequency, whereas spatially inhomogeneous strain broadens the Raman linewidth. In contrast, atomically flat hBN provides a van der Waals interface with weaker in-plane interactions, allowing the monolayer lattice to relax more effectively [30]. The resulting reduction in strain inhomogeneity accounts for the linewidth narrowing observed for Janus MoSSe and WSSe on hBN, supporting their improved uniformity and crystallinity.

**Figure 3.**
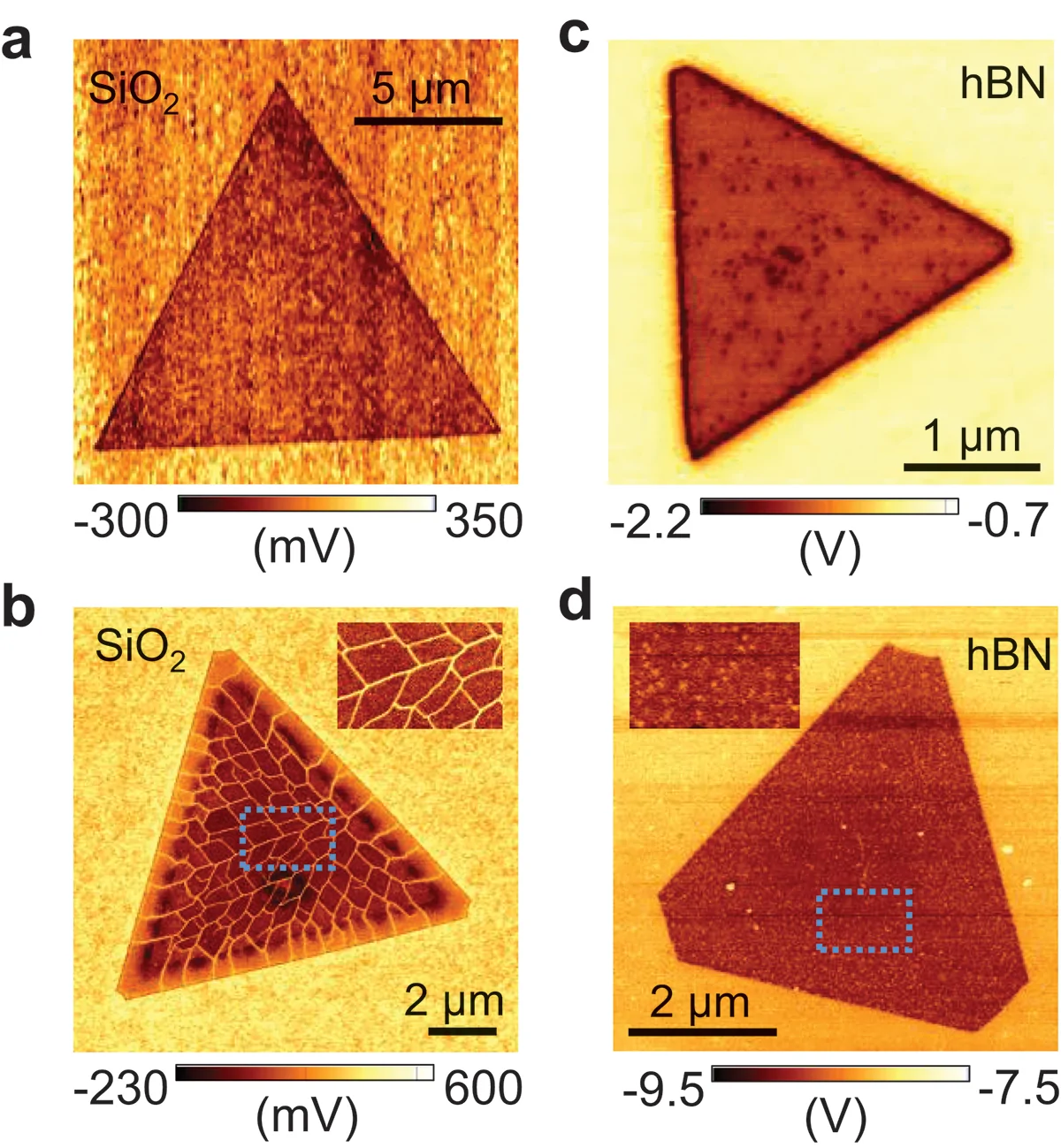
KPFM characterization of MoSe_2_ and Janus MoSSe monolayers. (a-d) KPFM surface-potential images and corresponding schematic illustrations of (a) MoSe_2_ monolayer on SiO_2_/Si, (b) Janus MoSSe monolayer on SiO_2_/Si, (c) MoSe_2_ monolayer on hBN, and (d) Janus MoSSe monolayer on hBN. Insets show enlarged images of the regions marked by the blue boxes. The low-potential dots in panel c are attributed to surface adsorbates and/or defects.

To examine the substrate dependence of crack formation during Janus conversion, we characterized the surface potential by KPFM. Figure 3(a–d) shows the surface-potential images of MoSe_2_ and Janus MoSSe monolayers prepared on SiO_2_ and hBN. The as-grown MoSe_2_ monolayers on both substrates exhibit crack-free surfaces (Figure 3(a,c)), indicating that cracking is not introduced during the initial CVD growth. In contrast, after Janus conversion, Janus MoSSe on SiO_2_ shows numerous cracks, whereas no cracks are observed for Janus MoSSe on hBN (Figure 3(b,d)). The cracks on SiO_2_ are readily identified in KPFM images by the pronounced surface-potential contrast at their edges. This crack formation on SiO_2_/Si can be explained by tensile strain generated when lattice contraction during Janus conversion is constrained by substrate pinning, whereas the weakly interacting hBN interface allows more efficient strain relaxation. These results highlight the critical role of atomically flat hBN in obtaining uniform, crack-free Janus TMD monolayers.

**Figure 4.**
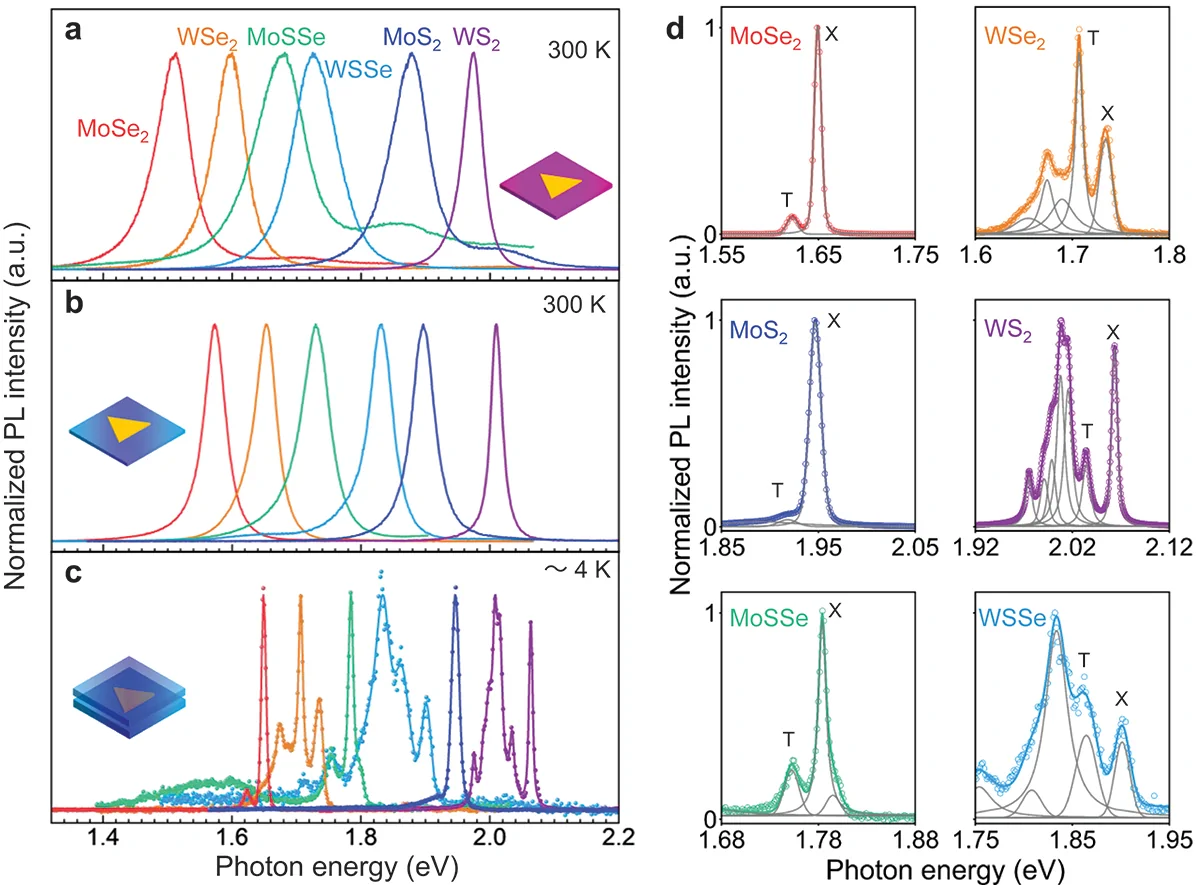
PL characterization. (a-c) PL spectra of MoSe_2_, WSe_2_, MoS_2_, WS_2_, Janus MoSSe, Janus WSSe monolayers prepared (a) on SiO_2_/Si and (b,c) hBN. The spectra were measured (a,b) at room temperature and (c) at around 4 K for hBN-encapsulated samples (hBN/TMD/hBN). (d) Voigt-function fits to the PL spectra in (c). X, neutral exciton; T, trion.

The improved optical uniformity is also confirmed by PL spectroscopy. Figure 4(a,b) shows the room-temperature PL spectra of monolayers of MoSe_2_, WSe_2_, MoS_2_, WS_2_, Janus MoSSe, and Janus WSSe prepared on SiO_2_/Si and hBN. The PL peak of Janus MoSSe (WSSe) is located between those of MoS_2_ and MoSe_2_ (WS_2_ and WSe_2_), reflecting the modified bandgap after one-sided chalcogen substitution, as observed previously [11–13]. This trend is consistent with the Raman-based assignment described above. The samples on hBN exhibit higher PL peak energies and narrower linewidths than those on SiO_2_/Si, consistent with reduced strain and suppressed bandgap fluctuations. The linewidths on hBN are 40, 43, 54, 45, 43, and 21 meV for MoSe_2_, WSe_2_, MoSSe, WSSe, MoS_2_, and WS_2_, respectively, compared with 63, 60, 90, 83, 67, and 37 meV on SiO_2_/Si. Statistical analysis further confirms that Janus MoSSe and WSSe on hBN exhibit narrower linewidths and higher PL peak energies than those on SiO_2_/Si (Figure S2, Supplementary Information). The linewidth reduction is attributed to the atomically flat hBN surface, which suppresses bandgap fluctuations associated with the surface roughness and interfacial disorder of SiO_2_/Si [38,39].

To probe low-temperature excitonic properties with minimal environmental disorder, we fabricated hBN-encapsulated samples (hBN/TMD/hBN). These samples exhibit well-resolved exciton and trion peaks (Figure 4(c)). To further examine the excitonic fine structure of Janus MoSSe and WSSe, we performed peak decomposition, as shown in Figure 4(d). The PL spectrum of Janus MoSSe resembles those of Mo-based TMDs (MoSe_2_ and MoS_2_): the neutral exciton (X) peak dominates, with a weaker trion (T) contribution. In addition to the neutral exciton and trion peaks, Janus WSSe exhibits multiple emission features similar to those observed in WSe_2_ and WS_2_ monolayers.

**Figure 5.**
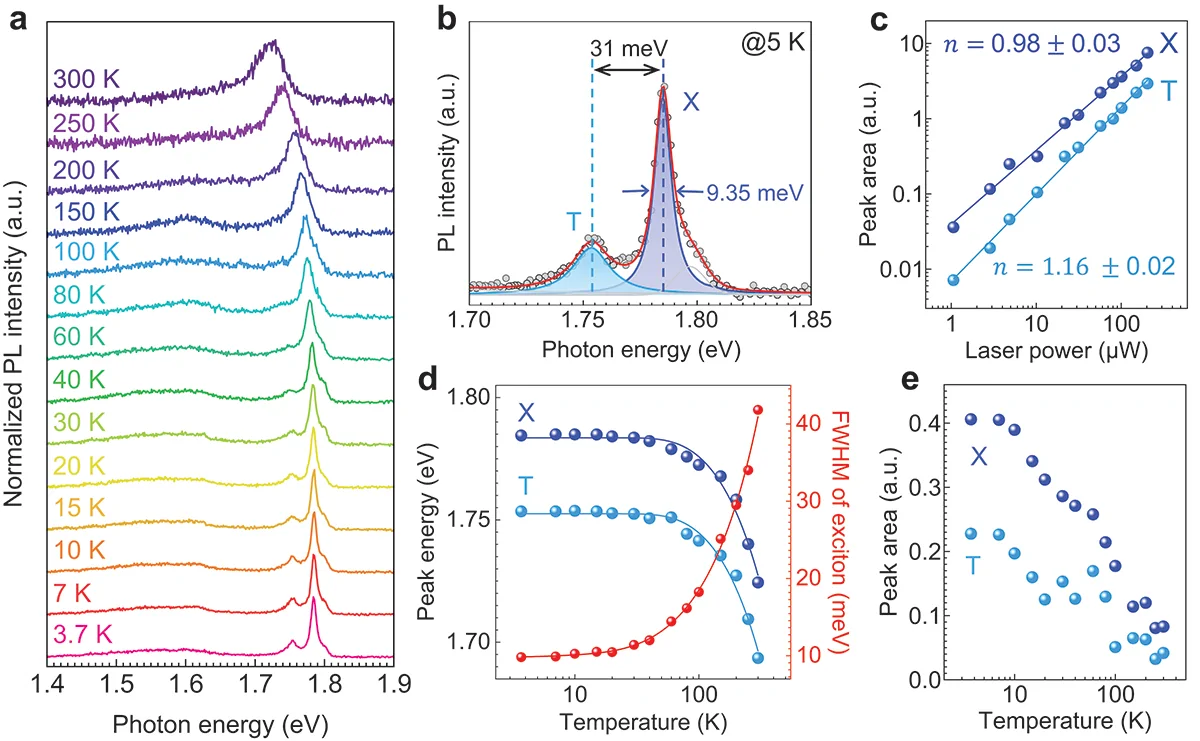
Temperature-dependent PL characterization of Janus MoSSe. (a) Temperature-dependent PL spectra of hBN-encapsulated Janus MoSSe monolayer (hBN/MoSSe/hBN). (b) Voigt-function fits to the PL spectrum at 5 K. (c) Excitation-power dependence of the integrated PL intensities of the neutral exciton (X) and trion (T) at 3.7 K. (d) Temperature dependence of the peak energies and FWHM of the neutral exciton, and the peak energies of the trion. (e) Temperature dependence of the integrated PL intensities of the neutral exciton and trion. The solid lines in (b-d) represent the fitting results.

To further investigate the excitonic properties and exciton-phonon interactions, we measured the temperature-dependent PL spectra of hBN-encapsulated Janus MoSSe and WSSe. For Janus MoSSe, the PL peak blueshifts and the linewidth decreases upon cooling, reflecting reduced exciton-phonon coupling at low temperature (Figure 5(a)). The exciton FWHM reaches 9.8 meV at 3.7 K, and an additional peak assigned to the trion appears on the lower-energy side of the exciton peak below 60 K. A PL spectrum measured at another position also exhibits two sharp peaks, with an exciton FWHM of 9.35 meV at 5 K (Figure 5(b)). To the best of our knowledge, this value is among the narrowest FWHMs reported for Janus MoSSe [13,15,17], indicating the high optical quality of the present Janus MoSSe prepared on the hBN. The trion binding energy, estimated from the energy separation between the exciton and trion peaks, is ~31 meV. To validate these assignments, we examined the excitation-power dependence of the integrated PL intensities at 3.7 K (Figure 5(c)). The peak areas follow a power-law dependence, $I\propto P^{n}$, with *n* = 0.98 ± 0.03 for the neutral exciton and *n* = 1.16 ± 0.02 for the trion. The nearly linear scaling of exciton emission indicates the absence of pronounced saturation in this power range, whereas the slightly superlinear behavior of the trion emission may reflect enhanced trion formation under increased excitation, possibly through photoinduced changes in carrier density.

The exciton and trion peak energies are plotted as functions of temperature in Figure 5(d). The temperature dependence was fitted using the O’Donnell–Chen model, which enables the extraction of the exciton-phonon coupling strength and average phonon energy [15,40]:

$$E\left(T\right)=E\left(0\right)-S\langle \hbar \omega \rangle \left[\coth\left(\frac{\left\langle \hbar \omega \right\rangle }{2k_{B}T}\right)-1\right],$$

where $k_{B}$ is the Boltzmann constant, $E\left(0\right)$ is the zero-temperature transition energy, S is a dimensionless exciton-phonon coupling constant, and ⟨ℏω⟩ is the average phonon energy. The fit yields $E\left(0\right)=$ 1.784 ± 0.002 eV, S= 1.6 ± 0.2, and ⟨ℏω⟩= 19 ± 4 meV for the neutral exciton. For the trion, the corresponding values are $E\left(0\right)=$ 1.753 ± 0.002 eV, S= 1.6 ± 0.2, and ⟨ℏω⟩= 18 ± 4 meV. The similar *S* and ⟨ℏω⟩ values for the exciton and trion suggest comparable coupling to thermally populated phonons.

The temperature dependence of the exciton FWHM is also fitted using the following equation:

$$\gamma =\gamma_{0}+c_{1}T+\frac{c_{2}}{\exp\left(\frac{\left\langle \hbar \omega \right\rangle }{k_{B}T}\right)-1},$$

where, $\gamma_{0}$ is the linewidth at 0 K, $c_{1}$ is the exciton-acoustic phonon scattering rate, and $c_{2}$ is the strength of the exciton-optical phonon scattering [17,41–43]. The average phonon energy ⟨ℏω⟩ is fixed to the value described above. The fit yields $\gamma_{0}=$ 9.2 ± 0.3 meV, $c_{1}=0.0$ 8 ±0.01 meV/K, and $c_{2}=$ 8 ± 3 meV. These values differ from a previous report on hBN-encapsulated Janus MoSSe ($c_{1}=$ 24 ± 5 µeV/K, $c_{2}=$ 124 ± 2 meV) [17]. We note that the extracted $c_{2}$ is sensitive to the choice of ⟨ℏω⟩ [43]. Reduced extrinsic broadening enables more direct evaluation of intrinsic exciton–phonon scattering.

We next examine how thermal population redistribution among exciton states affects the PL intensities. Figure 5(e) shows the temperature-dependent integrated PL intensities of the exciton and trion peaks, both of which decrease with increasing temperature. Because of conduction-band spin splitting, excitons can form bright (spin-allowed) and dark (spin-forbidden) states [44]. In Mo-based TMDs, such as MoS_2_ and MoSe_2_, the bright exciton is the lowest-energy exciton state. However thermal activation can redistribute the exciton population to optically inactive states, including dark exciton and nonradiative states, thereby reducing the bright-exciton PL intensity. This behavior is consistent with our results for Janus MoSSe. In contrast, Janus WSSe shows the opposite trends as shown below.

**Figure 6.**
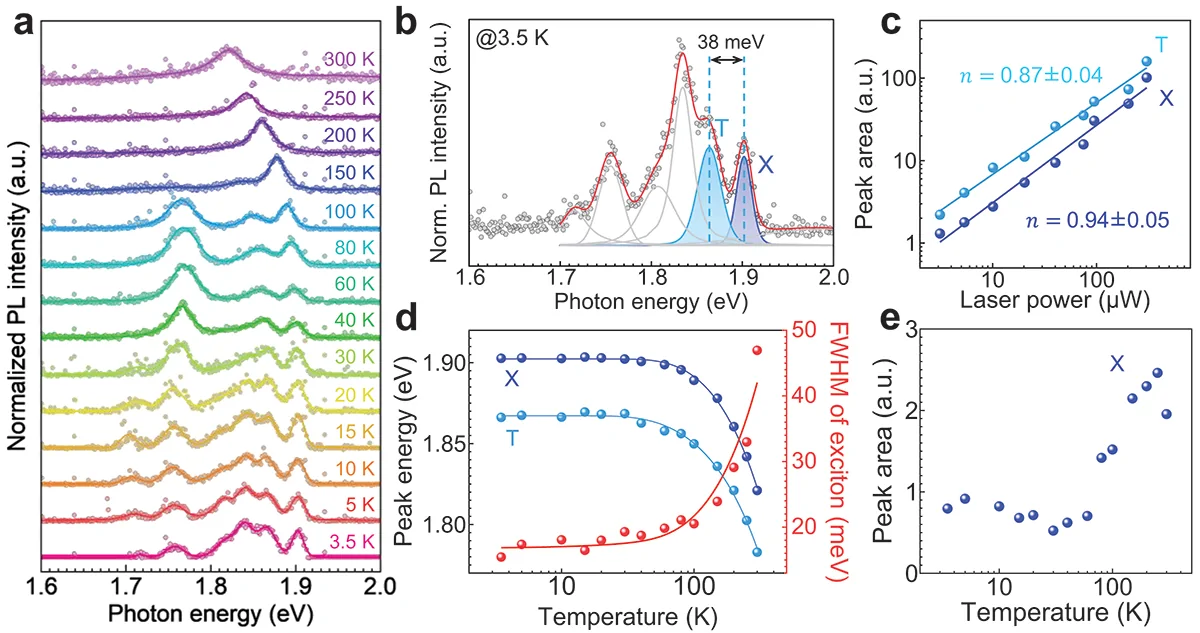
Temperature-dependent PL characterization of Janus WSSe. (a) Temperature-dependent PL spectra of hBN-encapsulated Janus WSSe monolayer (hBN/WSSe/hBN). (b) Voigt-function fits to the PL spectrum at 3.5 K. (c) Excitation-power dependence of the integrated PL intensities of the neutral exciton (X) and trion (T) at 3.5 K. (d) Temperature dependence of the peak energies and FWHM of the neutral exciton, and the peak energies of the trion. (e) Temperature dependence of the integrated PL intensities of the neutral exciton. The solid lines in (b-d) represent the fitting results.

For the Janus WSSe, temperature-dependent peak shifts and linewidth narrowing are similar to those of Janus MoSSe (Figure 6(a)). Upon cooling, the PL peak blueshifts and the linewidth decreases. However, the linewidth remains ~15 meV at 3.5 K, which is broader than the previously reported value of 5.9 meV [19]. This suggests that further improvement of the sample quality, such as reducing the defect density, may be important for Janus WSSe.Trion binding energy is estimated to be ~38 meV (Figure 6(b)), and the peak areas follow a power-law dependence with *n* = 0.94 ± 0.05 for the neutral exciton and *n* = 0.87 ± 0.04 for the trion (Figure 6(c)). At lower excitation powers, lower-energy peaks, possibly originating from localized states, dominate the PL spectrum (Figure S3, Supplementary Information). With increasing excitation power, these localized-state peaks gradually saturate, while the exciton and trion peaks become dominant. The additional PL peaks can be assigned to dark excitons and many-body excitonic complexes, reflecting the characteristic excitonic fine structure of W-based TMD monolayers [19,45]. More detailed assignment of these peaks will require measurements with carrier-density modulation. For the peak position and FWHM, the fitting results show $E_{G}\left(0\right)=$ 1.902 ± 0.002 eV, S= 2.26 ± 0.08, ⟨ℏω⟩= 19 ± 1 meV, $\gamma_{0}=$ 17 ± 1 meV, $c_{1}=$ 0.03 ± 0.03 meV/K, and $c_{2}=19\pm 10$ meV for exciton. For the trion, the corresponding values are $E\left(0\right)=$ 1.867 ± 0.002 eV, S= 1.96 ± 0.08, and ⟨ℏω⟩= 18 ± 4 meV. These values show trends similar to those of Janus MoSSe. However, the temperature dependence of the PL intensity is opposite to that of Janus MoSSe. The neutral-exciton PL intensity of Janus WSSe increases with increasing temperature. This behavior is characteristic of W-based TMD monolayers and can be attributed to thermal population transfer from lower-lying dark exciton states to the bright exciton state [44], suggesting that Janus WSSe retains an electronic structure similar to that of WSe_2_.

Finally, we discuss the role of hBN during the plasma-assisted chalcogen-substitution process and its impact on the optical quality of Janus TMDs. The present results show that hBN-supported chalcogen substitution suppresses extrinsic disorder during Janus conversion through weak mechanical coupling at van der Waals interface between hBN and TMDs. In particular, controlling strain is critical for fabricating Janus TMDs via chalcogen substitution, because lattice constant changes during substitution can readily generate strain. The reduced strain inhomogeneity, suppressed crack formation, and narrowed PL linewidths indicate that hBN enables both improved structural and optical uniformity of Janus TMD monolayers. This improved quality enables clearer access to intrinsic excitonic features, including trions, dark excitons, and many-body excitonic complexes, and allows quantitative analysis of exciton-phonon interactions. Therefore, the present strategy provides a useful platform for studying dipole-driven exciton physics and developing moiré and nanostructured Janus systems.

## 4. Conclusion

We demonstrated the preparation of high-quality Janus MoSSe and WSSe monolayers by combining CVD growth on hBN with room-temperature plasma-assisted chalcogen substitution. Compared with samples prepared on SiO_2_/Si, Janus MoSSe on hBN exhibits blueshifted and significantly narrower PL features, indicating suppressed inhomogeneous lattice strain. At room temperature, the PL linewidth reaches 54 meV, while the Raman linewidth narrows to 3.4 cm^−1^. KPFM measurements further reveal that crack formation during Janus conversion is suppressed on hBN, highlighting the critical role of the van der Waals interface in accommodating lattice contraction associated with atomic substitution. At low temperature, the PL linewidth narrows to 9.35 meV at 5 K for Janus MoSSe, supporting the high optical quality. Overall, our approach provides a general route to crack-free, low-strain Janus TMD monolayers with high optical quality, enabling detailed studies of intrinsic properties and phenomena arising from the built-in out-of-plane electric field. These high-quality Janus monolayers can also serve as versatile building blocks for designing in-plane and van der Waals heterostructures [46], nanoscrolls [25–27], and moiré superlattices [24].

## Supplementary Material

Supplemental Material
