# Supplementary material for: High-quality Janus MoSSe and WSSe monolayers enabled by hBN-supported chalcogen substitution
Source: Sci Technol Adv Mater. 2026 Aug 7;27(1):2713989. doi: 10.1080/14686996.2026.2713989 (PMC13591999; doi:10.1080/14686996.2026.2713989)
Supplement: Supplementary file 1 [file TSTA_A_2713989_SM8994.pdf]

Supplementary material

High-quality Janus MoSSe and WSSe monolayers enabled by hBN-supported chalcogen substitution

Tomoya Ogawa[a], Wenjin Zhang[b]*, Takahiko Endo[b], Yuta Sawai[a,b], Dingkun Bi[c,d], Tianyishan Sun[c,d], Hiroto Ogura[c,d], Kenji Watanabe[e], Takashi Taniguchi[b], Toshiaki Kato[c,d]*, and Yasumitsu Miyata[b,a]*

[a]*Department of Physics, Tokyo Metropolitan University, Hachioji, 192-0397, Japan*

[b]*Research Center for Materials Nanoarchitectonics (MANA), National Institute for Materials Science (NIMS), Tsukuba, 305-0044, Japan*

[c]*Advanced Institute for Materials Research (AIMR), Tohoku University, Sendai, 980-8577, Japan*

[d]*Graduate School of Engineering, Tohoku University, Sendai, 980-8579, Japan*

[e]*Research Center for Electronic and Optical Materials, NIMS, Tsukuba, 305-0044, Japan*

***Corresponding Authors**: miyata.yasumitsu@nims.go.jp; kato12@tohoku.ac.jp and zhang.wenjin@nims.go.jp

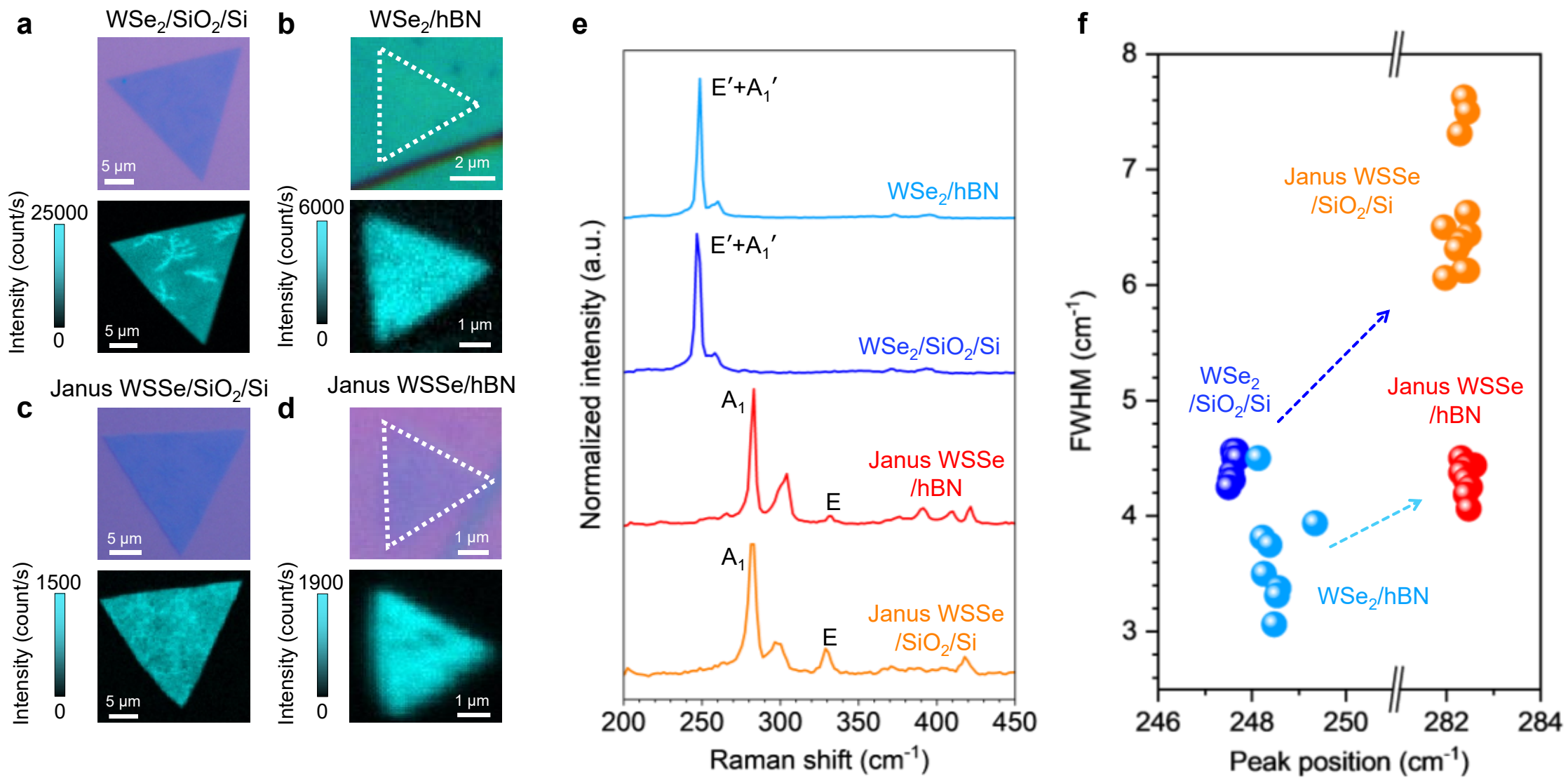

Figure S1. Raman characterization of WSe_2 and Janus WSSe monolayers. (a-d) Optical micrographs and corresponding Raman intensity maps of (a,b) the $E'+A_1'$ modes of WSe_2 and (c,d) the A_1 mode of Janus WSSe on SiO_2/Si and hBN. (e) Representative Raman spectra of WSe_2 and Janus WSSe monolayers on SiO_2/Si and hBN. (f) Distributions of the FWHM and peak positions of the $E'+A_1'$ modes of WSe_2 and the A_1 mode of Janus WSSe on SiO_2/Si and hBN.

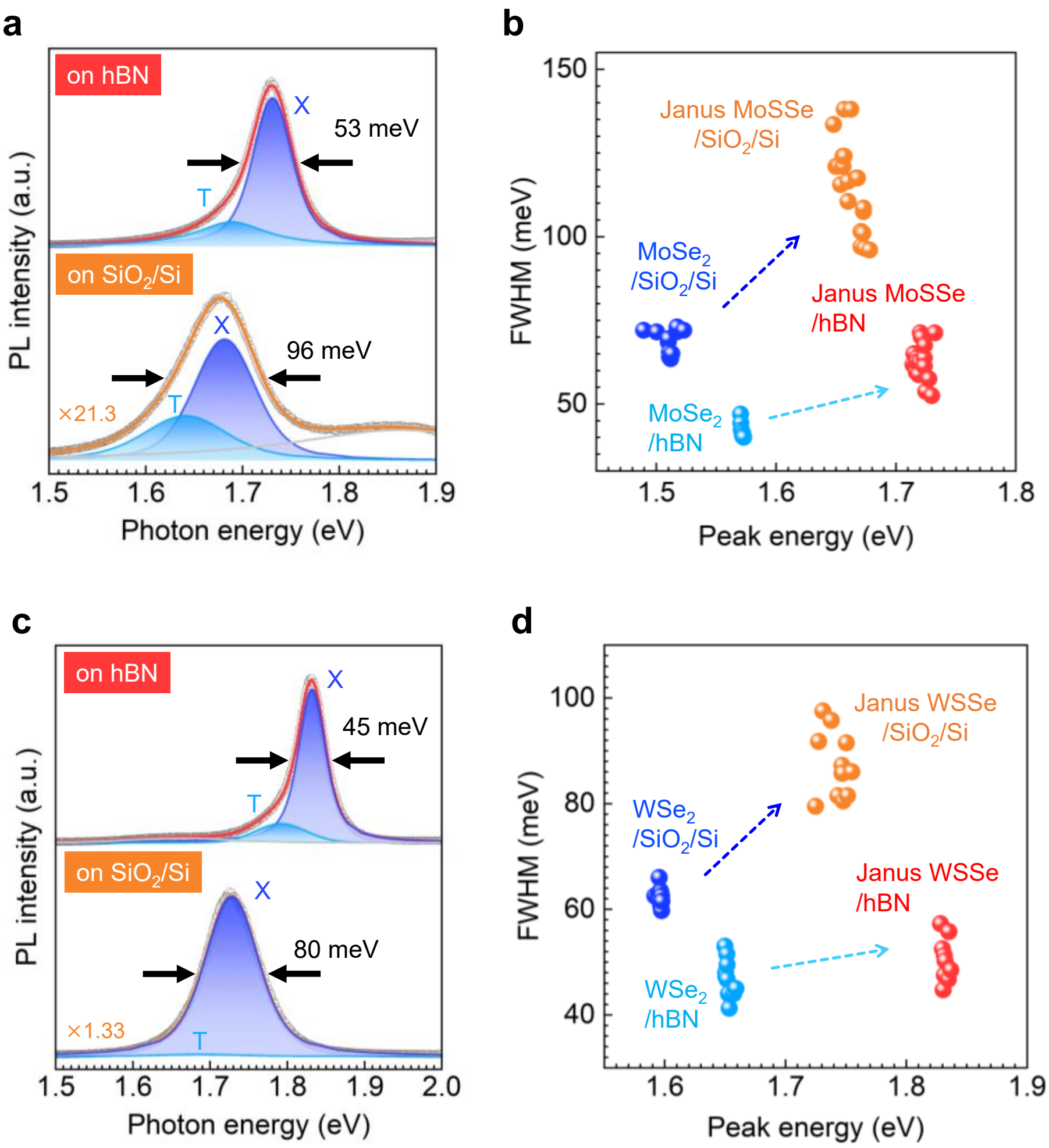

Figure S2. Room-temperature PL characterization of $MoSe_2$, WSe_2, Janus MoSSe, and Janus WSSe. (a) PL spectra of monolayer Janus MoSSe on SiO_2/Si and hBN. (b) Statistical distribution of FWHM and PL peak energy for monolayer $MoSe_2$ and monolayer Janus MoSSe on SiO_2/Si and hBN. (c) PL spectra of monolayer Janus WSSe on SiO_2/Si and hBN. (d) Statistical distribution of FWHM and PL peak energy for monolayer WSe_2 and monolayer Janus WSSe on SiO_2/Si and hBN. The FWHM was obtained for the exciton peak by fitting the PL spectra with exciton and trion components.

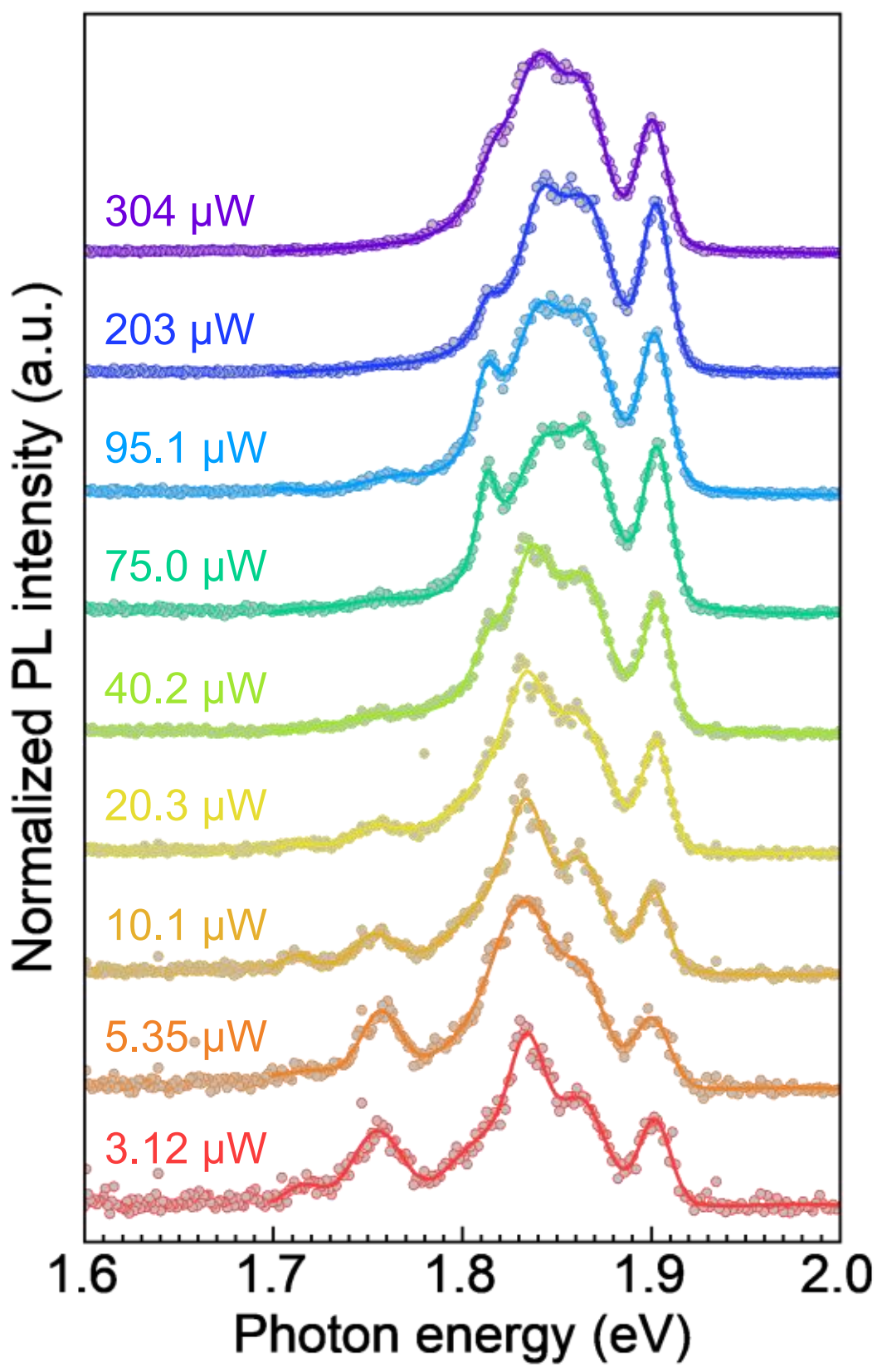

Figure S3. Excitation-power-dependent PL spectra of Janus WSSe. PL spectra of an hBN-encapsulated Janus WSSe monolayer measured at 3.5 K under different excitation laser powers.
